# Multi-omics reveal soil microbial dysbiosis and metabolite toxicity as drivers of blueberry continuous cropping obstacles

**DOI:** 10.3389/fmicb.2026.1880203

**Published:** 2026-06-26

**Authors:** Jiale Huang, Roland Bol, Dingrui Liu, Emeliane Kiladze, Xin Lou, Hexin Wang, Jiuming Zhang, Zhuang Ge, Tianhao Wang

**Affiliations:** 1 College of Life and Health, Dalian University, Dalian, China; 2 Institute of Bio- and Geosciences, Agrosphere (IBG-3), Forschungszentrum Jülich GmbH, Jülich, Germany; 3 Institute of Bio- and Geosciences, Plant Sciences (IBG-2), Forschungszentrum Jülich GmbH, Jülich, Germany; 4 Key Laboratory of Black Soil Protection and Utilization, Ministry of Agriculture and Rural Affairs, Heilongjiang Academy of Black Soil Conservation and Utilization, Heilongjiang Academy of Agricultural Sciences, Harbin, China

**Keywords:** autotoxicity, microbial diversity, rhizosphere interactions, soil metabolites, soil microbiome

## Abstract

Blueberry (*Vaccinium* spp.) are one of the most economically important fruit trees globally. However, due to continuous cropping have limited the industry’s ability to produce consistently over the long term, and the mechanism underlying the development of this continuous cropping problem is not yet fully understood. In this study, we applied metagenomic and metabolomic to systematically detect changes in microbial community structure, function and metabolic profiles in rhizosphere and non-rhizosphere soils after different years of continuous blueberry cultivation (0, 2, 4, and 6 years) in Dalian (China). The results showed that continuous cultivation significantly reduced overall microbial diversity and the bacterial and fungal Shannon index, with the decrease being more significant in the rhizosphere soils (*P* < 0.05). The β diversity analysis showed that the microbial community structure was distinctly separated between cultivation periods, with the most prominent differences in the rhizosphere soils (PERMANOVA, *P* < 0.01). The increased cultivation duration led to a decrease in the relative abundance of beneficial functional taxa in the microbial community, while the depletion-tolerant and stress-adapted taxa were gradually enriched. Functional annotation analysis showed that KEGG pathways related to stress response, amino acid degradation, and energy metabolism significantly increased, while functions related to nutrient transformation and plant-microbe interactions were weakened (FDR < 0.05). The metabolomic results further showed that 6 years of continuous cultivation significantly reshaped the rhizosphere metabolite composition. This was evidenced by the accumulation of various secondary metabolites in the rhizosphere soil, including metabolites related to potential self-toxicity (e.g., ferulic acid, 3-hydroxyphenylacetic acid, and 2-hydroxycinnamic acid), mainly involved in the pathways of amino acid metabolism, lipid metabolism, and secondary metabolite synthesis. In conclusion, continuous cultivation of blueberry induced pronounced shifts in rhizosphere microbial community structure, function, and metabolite composition, suggesting that these changes may contribute to the development of continuous cropping obstacles (CCO).

## 1 Introduction

Blueberries (*Vaccinium* spp.) belong to the Ericaceae family, and their commercial cultivation began in the early 20th century (Ratnaparkhe, 2007). Blueberries have rapidly expanded globally to cover North America, South America, Europe, and Asia, with China experiencing particularly rapid growth (Duan et al., 2022).

Unlike annual crops, which are typically grown in succession, blueberries are perennial crops that remain in the same soil for many years. This prolonged soil occupancy makes blueberries more susceptible to the negative effects of continuous cropping, which leads to suppressed crop growth, reduced yield and quality, and increased incidence of soil-borne diseases when the same or related crops are repeatedly cultivated on the same land (Ma Z. et al., 2023). With the widespread adoption of continuous cropping driven by limited arable land, CCO have been well documented in a wide range of agricultural systems (Liu Y. et al., 2024). In perennial cropping systems, the negative effects of continuous monoculture tend to accumulate over time and are often difficult to reverse through short-term management practices (Li Q. et al., 2023). For example, perennial fruit trees such as apples, pears, cherries, and peaches exhibit pronounced replant disease (i.e., continuous cropping obstacles) during long-term monoculture. The direct cause is that their root systems remain in the same soil for extended periods, leading to the gradual accumulation of soil-borne pathogens and harmful metabolites. Meanwhile, the plants’ resistance declines due to repeated annual fruiting, ultimately resulting in restricted root growth and reduced yield. As a perennial fruit crop, blueberry similarly lacks a mechanism to reset its rhizosphere environment during its growth cycle, and therefore also experiences comparable continuous cropping obstacles (Ajeethan et al., 2023). However, the mechanisms underlying these negative effects have not yet been fully elucidated.

There is growing evidence that CCO arise from complex multifactorial processes, and that biological processes mediated by changes in the structure of soil microbial communities and their functional potential play an important role in the formation of CCO (Li Q. et al., 2023). Long-term continuous monoculture has been widely reported to significantly alter the structure and diversity of soil microbial communities and their functional potential (Guo et al., 2014; Li W. et al., 2023; Wang et al., 2025; Li et al., 2026). Continuous cropping promotes the enrichment of soil-borne pathogenic microorganisms while causing a decrease in beneficial microorganisms (Wang et al., 2024). Notably, pathogen enrichment under continuous cropping is frequently crop-specific, with crop-specific pathogens reported in different systems (Lv et al., 2020; Wu et al., 2023).

In addition to microbial changes, continuous cropping systems are often accompanied by significant alterations in the composition of soil metabolites, a process that has been gradually recognized as one of the most important influences in the formation of CCO (Xu et al., 2025). Under long-term monocropping conditions, specific chemosensitizers (especially phenolic acids) tend to accumulate in the soil due to insufficient degradation capacity and cause autotoxic effects (Song et al., 2024). In addition, a growing body of research suggests that these metabolites may further exacerbate the occurrence of microbial imbalances in continuous cropping systems by modulating the soil microbial environment (Chaparro et al., 2012; Liu Y. et al., 2024). However, the relative importance of these factors and their interactions remain inconsistent across studies. Although microbial changes and metabolite accumulation are increasingly recognized as important contributors to CCO, their dynamics and specific roles still vary under different continuous cropping conditions, highlighting the need for systematic research.

In particular, the effects of different years of continuous cropping and rhizosphere and non-rhizosphere soil differences in blueberry systems remain understudied. Therefore, combining the results of existing research on the CCO of other crops, long-term continuous cropping of blueberries may cause significant changes in soil microbiota. Based on the above hypotheses, the present study used soil metagenomics and metabolomics to characterize the soil biology in different years of continuous cropping and different rhizosphere soil environments under continuous blueberry cultivation. We hypothesized: (i) the structure and functional composition of the soil microbial community may change with increasing years of continuous cropping, as evidenced by a decrease in beneficial microorganisms and a gradual increase in potentially pathogenic microorganisms; (ii) long-term continuous cropping may lead to changes in the metabolite composition of blueberry soils, and some secondary metabolites thought to be associated with autotoxicity may accumulate in soils; and (iii) the above microbial and metabolic changes may differ between rhizosphere and non-rhizosphere soils, affecting the formation of blueberry CCO in different dimensions.

## 2 Materials and methods

### 2.1 Experimental site and soil sampling

Soil samples were collected from a blueberry orchard in Dalian, China. The soil type was Alfisols (The basic physical and chemical data for the soil have been added to Supplementary Table 1), and the site was located at 39.29°N latitude and 122.05°E longitude. The area has a warm-temperate semi-humid monsoon climate with an average annual temperature of about 10°C and annual precipitation of 550–950 mm. Sampling was carried out on August 8, 2024. The experimental material used in this study was “Liberty,” a cultivar of northern highbush blueberry. Ammonium sulfate was applied biweekly from May to July at a rate of 37.5–45 kg ha^–1^, consistent management of all processes. In order to investigate the effects of different cropping durations and rhizosphere conditions on blueberry CCO, six soil types with distinct cultivation histories were selected for this study. These included a healthy control soil not previously planted with blueberries (CK), soils subjected to 2, 4, and 6 years of continuous blueberry cropping (CC2, CC4, and CC6), and rhizosphere soils collected under 4 and 6 years of continuous cropping conditions [CC4 (RH) and CC6 (RH)].

In the CC4 and CC6 groups, both rhizosphere soils [CC4 (RH), CC6 (RH)] and non-rhizosphere soils (CC4, CC6) were sampled, whereas in the CK and CC2 groups, only non-rhizosphere soils were collected. Rhizosphere soils were obtained by gently shaking the root system to remove loosely attached soil, followed by brushing off the soil tightly adhering to the root surface with a sterile bristle brush. Non-rhizosphere soils were taken from soil blocks not directly affected by the root system. Each plot was sampled at a depth of 0–20 cm using a five-point composite sampling method, with subsamples randomly collected and thoroughly homogenized to form representative composite samples. Each composite sample was considered as one independent biological replicate, and three biological replicates were collected for each group. The collected soil samples were transported to the laboratory in sterile polyethylene bags and stored at −80°C for microbial DNA extraction and metabolite analysis.

### 2.2 Metagenomic sequencing and data processing

Total genomic DNA was extracted from 0.2 g of rhizosphere and non-rhizosphere soils using the E.Z.N.A.^®^ Soil DNA Kit (Omega Bio-Tek, United States) according to the manufacturer’s instructions. DNA concentrations were monitored by a microfluorometer (TBS-380, Turner Biosystems, United States) and sequenced using a sequencing kit (NovaSeq Reagent Kits, Illumina, United States) for metagenomic sequencing on an Illumina NovaSeq™ X Plus platform at Majorbio Bio-Pharm Technology Co., Ltd. (Shanghai, China). Raw sequencing data have been deposited in the NCBI Sequence Read Archive (SRA) under the BioProject accession number PRJNA1404893.

Briefly, raw reads were trimmed to remove adapters and low-quality sequences (length < 50 bp or average quality value < 20) using fastp (version 0.20.0).^1^ Quality-filtered reads were assembled using MEGAHIT (version 1.1.2),^2^ and contigs ≥ 300 bp were retained. Open reading frames (ORFs) were predicted with Prodigal (version 2.6.3),^3^ and ORFs ≥ 100 bp were selected. A non-redundant gene catalog was constructed using CD-HIT (version 4.7)^4^ with 90% sequence identity and 90% coverage. Gene abundance for each sample was estimated by mapping reads to the catalog using SOAPaligner (version 2.21)^5^ with 95% identity. Taxonomic annotation of non-redundant genes was performed by aligning sequences against the NCBI NR database using DIAMOND (version 2.0.13)^6^ with an e-value cutoff of 1e-5.

### 2.3 Soil metabolite extraction and LC-MS/MS analysis

For metabolite extraction, 100 mg of soil was placed in a 2 mL centrifuge tube with a 6 mm grinding bead, and 800 μL of extraction solution (methanol: water = 4:1, v/v, containing four internal standards, e.g., 0.02 mg/mL L-2-chlorophenylalanine) was added. Samples were ground using a frozen tissue grinder (Wonbio-96c, Shanghai Wanbo Biotechnology Co., Ltd.) at −10°C, 50 Hz for 6 min, followed by low-temperature ultrasonic extraction for 30 min (5°C, 40 kHz). After incubation at −20°C for 30 min, samples were centrifuged (13,000 g, 4°C, 15 min), and the supernatant was transferred to injection vials for LC-MS/MS analysis.

A pooled quality control (QC) sample was prepared by mixing equal volumes of all samples and injected periodically (every 5–15 samples) to monitor instrument stability.

LC-MS/MS analysis was performed on a UHPLC-Orbitrap Exploris 240 system equipped with an ACQUITY HSS T3 column (100 mm × 2.1 mm i.d., 1.8 μm; Waters, United States) at Majorbio Bio-Pharm Technology Co., Ltd. (Shanghai, China). Mobile phases were 0.1% formic acid in water: acetonitrile (2:98, v/v, solvent A) and 0.1% formic acid in acetonitrile (solvent B), with a flow rate of 0.4 mL/min and column temperature of 40°C. The injection volume was 5 μL. The ESI source operated in positive and negative modes with a source temperature of 400°C, sheath gas 40 arb, auxiliary gas 10 arb, and ion-spray voltage of −2800 V (negative) and 3,500 V (positive). Data were acquired in Data-Dependent Acquisition (DDA) mode over an m/z range of 70–1,050, with MS/MS normalized collision energy of 20–40–60 V.

Raw LC-MS/MS data were preprocessed using Progenesis QI (Waters Corporation, Milford, United States) to generate a three-dimensional CSV matrix including sample information, metabolite name, and spectral intensity. Internal standards and known artifacts (noise, column bleed, derivatization peaks) were removed. Metabolites were annotated using HMDB,^7^ Metlin,^8^ and the in-house Majorbio Database (MJDB, Majorbio Biotechnology Co., Ltd.). The processed data matrix was uploaded to the Majorbio cloud platform^9^ for further analysis. Features detected in at least 80% of samples were retained; missing values were filled with the minimum value, and peak intensities were normalized to the sum. QC features with RSD > 30% were removed, and data were log10 transformed prior to variance analysis and downstream statistical evaluation.

### 2.4 Data processing

Soil bacterial and fungal communities were analyzed separately. α diversity was assessed by the Shannon and Simpson indices for within-community diversity, and β diversity was calculated based on Bray–Curtis distances and visualized by principal coordinate analysis (PCoA) to characterize differences in community structure. Community composition was summarized at the phylum and genus levels. Differences in abundance between groups were examined using a linear discriminant analysis effect size (LEfSe) approach, with significance determined by the Kruskal–Wallis rank-sum test and discriminant thresholds set at LDA > 3.0 for bacteria and > 4.0 for fungi, respectively. Microbial gene abundance was summarized at the KEGG secondary functional level. Multiple group comparisons at the KEGG Orthology (KO) level were performed using limma linear modeling with empirical Bayesian correction, whereas two-group comparisons were evaluated using the Wilcoxon rank-sum test with false discovery rate (FDR) correction. KOs with major functional changes were identified based on the results of the analysis of variance and functionally annotated via the KEGGREST database. Pathway-level differences were comprehensively assessed by the GRSA reporter score, combining log_2_ fold change of KOs and significance information to measure pathway enrichment trends; disease-related pathways were also filtered to highlight environmentally functionally relevant metabolic processes.

Metabolomic analyses were designed to reveal differences in metabolic functions between continuous and non-continuous soils. Metabolite abundance data were log-transformed and standardized to characterize between-group differences in overall metabolomic profiles by partial least squares discriminant analysis (PLS-DA). Significant differences in metabolites between the two groups were assessed using independent samples *t*-tests with false discovery rate (FDR) correction by the Benjamini-Hochberg method, and key metabolites were screened in conjunction with variable importance projection (VIP) values from the PLS-DA model. Significant metabolites were analyzed by hierarchical clustering to demonstrate their abundance distribution patterns among samples. Subsequently, differential metabolites were mapped to KEGG compound IDs and categorized into KEGG secondary functional categories. Pathway-level enrichment significance was assessed based on hypergeometric tests and enrichment ratio calculations to identify key metabolic functions associated with disrupted connectivity.

## 3 Results

### 3.1 Changes in soil microbial community diversity

**FIGURE 1:**
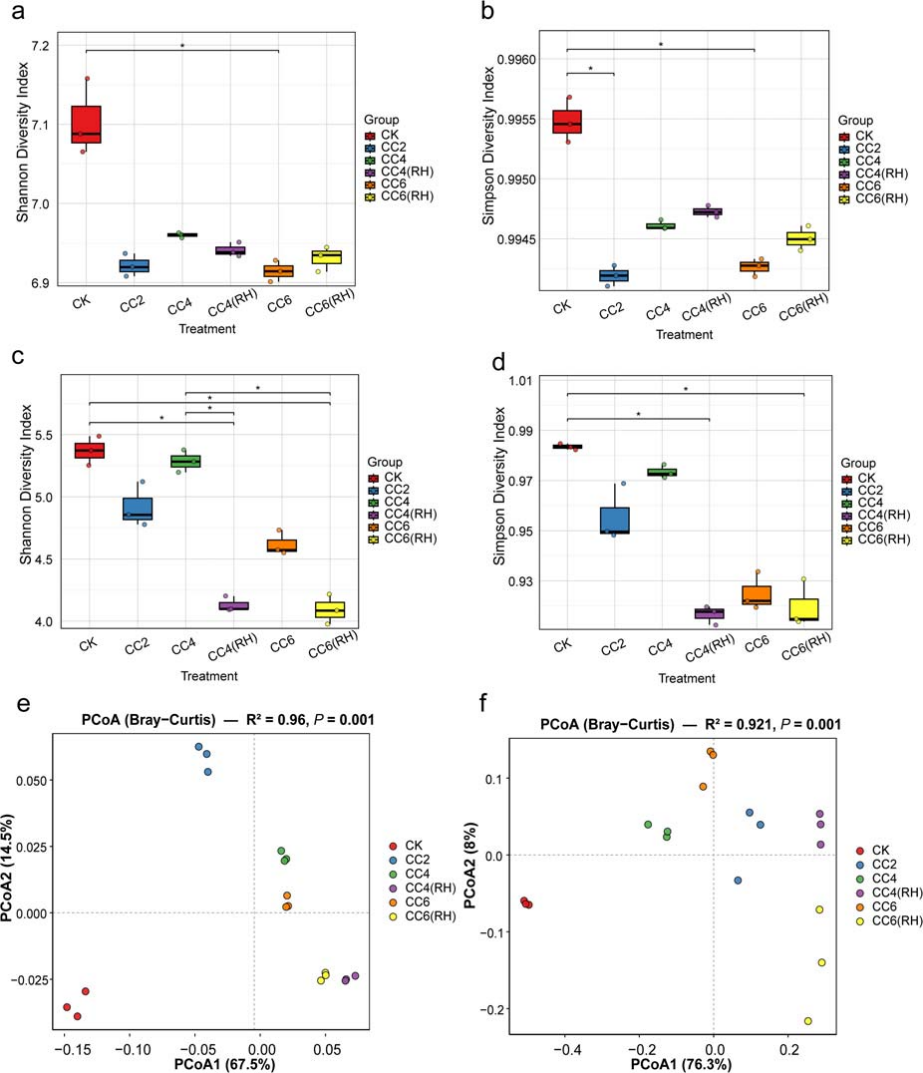
Alpha and beta diversity of soil microbiomes under different continuous-cropping durations. Boxplots of bacterial Shannon **(a)** and Simpson **(b)** indices, and fungal Shannon **(c)** and Simpson **(d)** indices. Asterisks indicate significant differences between groups (*P < 0.05). PCoA of bacterial communities (Bray–Curtis) **(e)**. PCoA of fungal communities (Bray–Curtis) **(f)**. CK, control soil without blueberry cropping; CC2, soil under 2-year continuous cropping; CC4, bulk soil under 4-year continuous cropping; CC4 (RH), rhizosphere soil under 4-year continuous cropping; CC6, bulk soil under 6-year continuous cropping; CC6 (RH), rhizosphere soil under 6-year continuous cropping.

The α-diversity of microbial communities (species level, Figures 1a–d) was assessed using multiple indices. The bacterial Shannon and Simpson indices were significantly lower in the CC6 group than in CK (*P* < 0.05), whereas the changes in CC4 and CC4 (RH) were not significant (Figures 1a,b). In contrast, Shannon and Simpson indices of fungi were significantly lower (*P* < 0.05) than those of CK in both CC6 (RH) and CC4 (RH), whereas the changes in CC6 and CC4 were not significant. Principal coordinate analysis (PCoA, genus level, Figures 1e,f) based on Bray-Curtis distances showed significant differences in microbial community structure among groups (bacteria: *R*^2^ = 0.96, *P* = 0.001; fungi: *R*^2^ = 0.921, *P* = 0.001).

### 3.2 Soil microbial community composition

**FIGURE 2:**
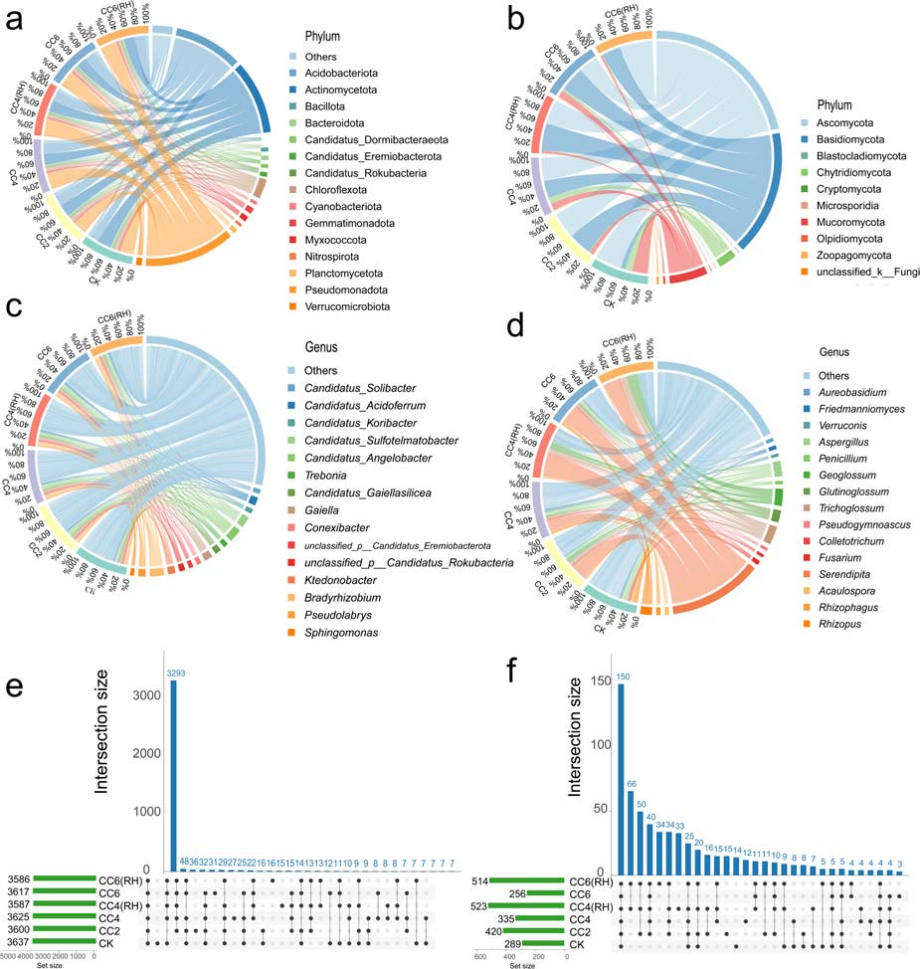
Taxonomic composition and distribution of soil bacterial and fungal communities across continuous-cropping durations. Circos plots showing the relative abundance of bacterial phyla **(a)**, fungal phyla **(b)**, bacterial genera **(c)**, and fungal genera **(d)**. UpSet plots showing shared and treatment-specific bacterial **(e)** and fungal **(f)** genera across the six soil treatments. CK, control soil without blueberry cropping; CC2, soil under 2-year continuous cropping; CC4, bulk soil under 4-year continuous cropping; CC4 (RH), rhizosphere soil under 4-year continuous cropping; CC6, bulk soil under 6-year continuous cropping; CC6 (RH), rhizosphere soil under 6-year continuous cropping.

At the phylum level, the bacterial community (Figure 2a) consisted mainly of Actinomycetota, Pseudomonadota, and Acidobacteriota, with a total abundance of more than 60%. Actinomycetota was highest in CK (27.8%) and lower in CC6 (20.0%) and CC6 (RH) (21.5%). Acidobacteriota was relatively stable across groups (17.9–22.6%). At the genus level (Figure 2c), most bacteria showed little change. The most changed genus was Candidatus Sulfotelmatobacter, whose relative abundance increased from CK (2.7%) to CC6 (4.0%) and CC6 (RH) (3.8%), but the change was still limited. The fungal communities differed significantly between CK and continuous cropping soils. The main phyla (Figure 2b) were Ascomycota and Basidiomycota, followed by Mucoromycota, but the proportions varied greatly. Ascomycota accounted for 48.0% in CK, decreased to 36.4% in CC6, and increased to 60.6% in CC6 (RH), and 48.1, 42.9, and 44.8% in CC2, CC4, and CC4 (RH), respectively. Basidiomycota was 8.7% in CK, increased to 45.5% in CC6, and decreased to 34.6% in CC6 (RH), and was 41.7, 31.2, and 49.9% in CC2, CC4, and CC4 (RH), respectively. Mucoromycota decreased from 26.3% in CK to 11.9% in CC6, and further decreased to 3.4% in CC6 (RH), and was 7.3, 11.8, and 4.2% in CC2, CC4, and CC4 (RH), respectively, indicating that this phylum was suppressed under continuous rhizosphere soil rooting.

At the genus level (Figure 2d), *Serendipita* was almost absent in CK (0.4%), increased to 36.6% in CC6, and further to 30.1% in CC6 (RH), and was 32.3, 21.5, and 44.3% in CC2, CC4, and CC4 (RH), respectively. *Geoglossum* and *Trichoglossum* were very low in CK (0.1 and 0.2%), increased to 2.6 and 3.0% in CC6, and further increased to 12.9% and 13.7% in CC6 (RH), and were 5.0, 1.9, 7.5 and 5.4%, 2.3, and 7.9% in CC2, CC4, and CC4 (RH), respectively. In contrast, *Aspergillus* decreased from 9.9% in CK to 5.5 and 1.5% in CC6 and CC6 (RH), and was 3.5, 5.1, and 1.5% in CC2, CC4, and CC4 (RH), respectively; *Rhizopus* decreased from 7.6% in CK to 4.6 and 0.9% in CC6 and CC6 (RH), and was 2.3, 4.2, and 0.9% in CC2, CC4, and CC4 (RH), respectively.

The UpSet plots show the distribution of shared versus endemic genera at the bacterial (Figure 2e) and fungal (Figure 2f) phylum level in the six soil groups. Bacteria had 3,293 genera present in all groups, while CK, CC2, CC4 (RH), CC6, and CC6 (RH) had 48, 29, 13, 25, and 14 endemic genera, respectively, with CC4 having a very low number of endemic genera, and therefore not shown in the plot. Fungi had 150 genera common to all groups, while CK, CC2, CC4, CC6, and CC6 (RH) had 14, 15, 12, 3, and 34 endemic genera, respectively. These results demonstrate the sharing of bacteria and fungi at the phylum level, with distinct distribution patterns among groups.

### 3.3 Differential microbial biomarker analysis

**FIGURE 3:**
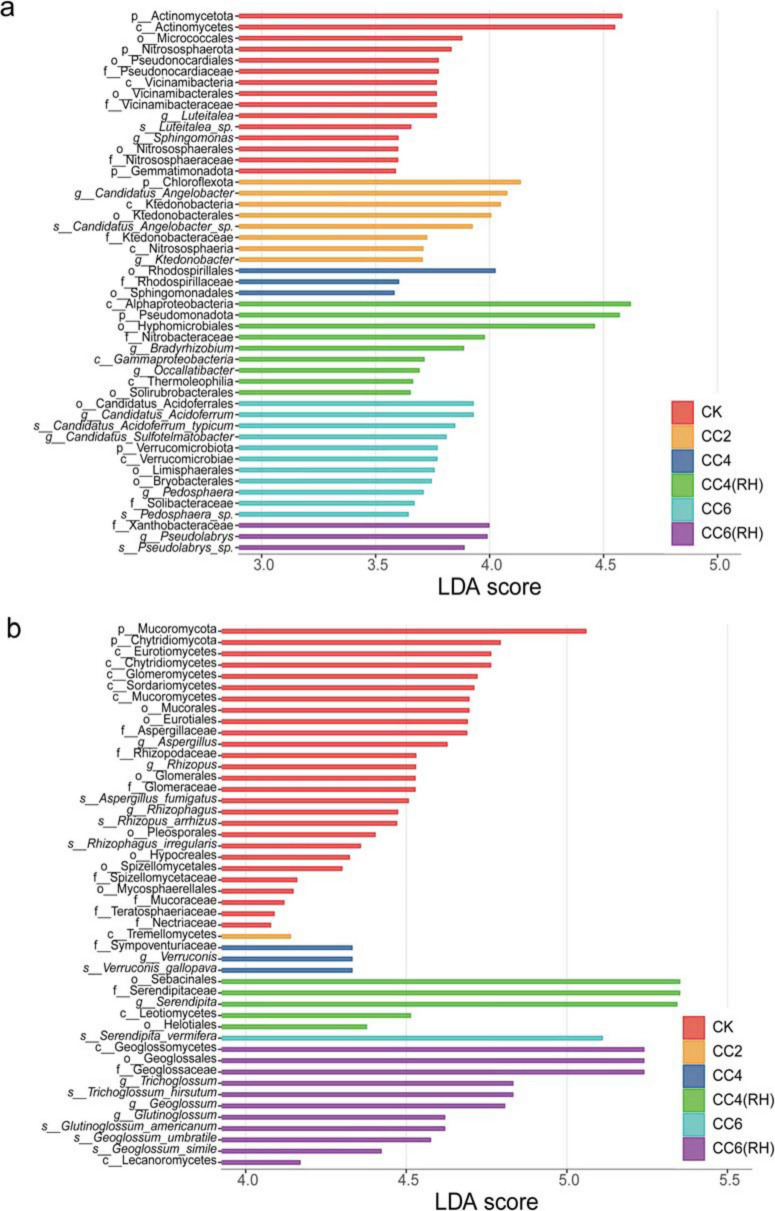
LEfSe-based identification of differential microbial biomarkers across soil treatments. LDA bar plots from LEfSe identifying significantly enriched bacterial taxa **(a**; LDA > 3.0, *P* < 0.05) and fungal taxa **(b**; LDA > 4.0, *P* < 0.05). CK, control soil without blueberry cropping; CC2, soil under 2-year continuous cropping; CC4, bulk soil under 4-year continuous cropping; CC4 (RH), rhizosphere soil under 4-year continuous cropping; CC6, bulk soil under 6-year continuous cropping; CC6 (RH), rhizosphere soil under 6-year continuous cropping.

LEfSe analysis (LDA > 3.0, *P* < 0.05) identified significant differences in bacterial taxa among the soil groups (Figure 3a). In the CK group, enriched taxa included the phylum Actinomycetota and its subordinate class Actinomycetes, orders Micrococcales and Pseudonocardiales, and the family Pseudonocardiaceae. The phylum Nitrososphaerota and its subordinate order Nitrososphaerales and family Nitrososphaeraceae were also enriched. In addition, the class Vicinamibacteria and its subordinate order Vicinamibacterales, family Vicinamibacteraceae, genus *Luteitalea*, and species *Luteitalea* sp. were enriched, together with the phylum Gemmatimonadota and the genus *Sphingomonas*. In the CC2 group, enrichment was observed for the phylum Chloroflexota and its subordinate class Ktedonobacteria, order Ktedonobacterales, family Ktedonobacteraceae, and genus *Ktedonobacter*, as well as the genus *Candidatus Angelobacter* and species *Candidatus Angelobacter* sp. The class Nitrososphaeria was also enriched. In the CC4 group, fewer taxa were detected, including the order Rhodospirillales and family Rhodospirillaceae, and the order Sphingomonadales. In the CC4 (RH) group, enriched taxa included the phylum Pseudomonadota and its subordinate classes Alphaproteobacteria and Gammaproteobacteria, as well as the order Hyphomicrobiales, family Nitrobacteraceae, and genus *Bradyrhizobium*. The class Thermoleophilia and its subordinate order Solirubrobacterales and genus *Occallatibacter* were also enriched in the CC6 group. Enriched taxa included the phylum Verrucomicrobiota and its subordinate class Verrucomicrobiae, order Limisphaerales, genus *Pedosphaera*, and species *Pedosphaera* sp. Additional enriched taxa were the order Candidatus Acidoferrales and its subordinate genus *Candidatus Acidoferrum* and species *Candidatus Acidoferrum typicum*, as well as the order Bryobacterales, family Solibacteraceae, and genus *Candidatus Sulfotelmatobacter*. In the CC6 (RH) group, enrichment was observed for the family Xanthobacteraceae and its subordinate genus *Pseudolabrys* and species *Pseudolabrys* sp.

LEfSe analysis (LDA > 4.0, *P* < 0.05) identified significant differences in fungal taxa among the soil groups (Figure 3b). In the CK group, the dominant fungal taxa were mainly affiliated with the phylum Mucoromycota, including the class Mucoromycetes (order Mucorales, family Mucoraceae, genus *Rhizopus*, species *Rhizopus arrhizus*) and the class Glomeromycetes (order Glomerales, family Glomeraceae, genus *Rhizophagus*, species *Rhizophagus irregularis*). The family Rhizopodaceae was also detected.

In addition, members of the phylum Chytridiomycota (class Chytridiomycetes, order Spizellomycetales, family Spizellomycetaceae), class Sordariomycetes (order Hypocreales, family Nectriaceae), class Eurotiomycetes (order Eurotiales, family Aspergillaceae, genus *Aspergillus*, species *Aspergillus fumigatus*), order Mycosphaerellales (family Teratosphaeriaceae), and order Pleosporales were also enriched. In the CC2 group, fungi belonging to the class Tremellomycetes were enriched. In the CC4 group, enriched taxa included the family Sympoventuriaceae (genus *Verruconis*, species *Verruconis gallopava*). In the CC4 (RH) group, enriched taxa included the class Leotiomycetes (order Helotiales) and the independent order Sebacinales (family Serendipitaceae, genus *Serendipita*). In the CC6 group, the species *Serendipita vermifera* was enriched. In the CC6 (RH) group, enriched taxa included the class Geoglossomycetes (order Geoglossales, family Geoglossaceae, genus *Geoglossum*, species *Geoglossum simile* and *Geoglossum umbratile*/genus *Glutinoglossum*, species *Glutinoglossum americanum*/genus *Trichoglossum*, species *Trichoglossum hirsutum*) and the class Lecanoromycetes.

### 3.4 KEGG functional profiling of soil microbial communities

**FIGURE 4:**
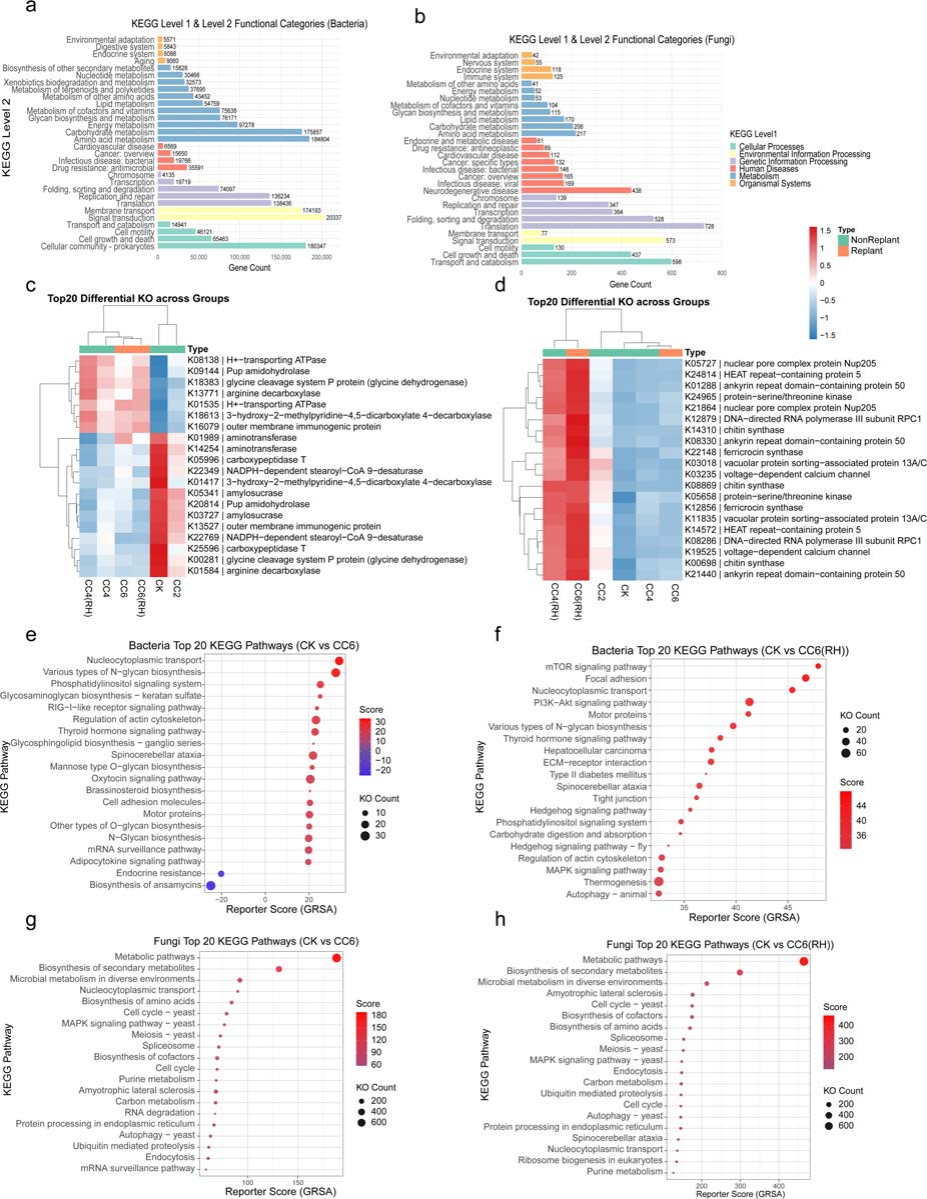
Functional profiling and KEGG pathway enrichment of soil bacterial and fungal communities under continuous cropping. KEGG Level 1 and Level 2 functional classifications forbacteria **(a)** and fungi **(b)**. Heatmaps showing the top 20 most differentially abundant KEGG Orthologs (KOs) for bacteria **(c)** and fungi **(d)**. KEGG pathway enrichment results for bacteria **(e,f)** and fungi **(g,h)** comparing CK with long-term continuous cropping soils. CK, control soil without blueberry cropping; CC2, soil under 2-year continuous cropping; CC4, bulk soil under 4-year continuous cropping; CC4 (RH), rhizosphere soil under 4-year continuous cropping; CC6, bulk soil under 6-year continuous cropping; CC6 (RH), rhizosphere soil under 6-year continuous cropping.

Further analyzing the microbial functions, after functional comparison in the KEGG database, the pathway with the highest number of genes annotated to KEGG pathway level 1 in bacteria (Figure 4a) was Metabolism, and all the genes annotated to KEGG pathway level 2 were mainly carbohydrate metabolism, amino acid metabolism, and energy-related pathways. The difference in the number of genes annotated to KEGG pathway level 1 in fungi (Figure 4b) was small, and the main pathways annotated to KEGG pathway level 2 were Transport and Catabolism, Translation, and Signal Transduction.

Heatmap of bacterial KEGG KOs (Figure 4c) showed that the most abundant KOs in CK soils were mainly attributed to basal metabolic functions, including amino acid interconversion (aminotransferase, glycine cleavage system P protein), protein turnover (carboxypeptidase T, Pup amidohydrolase), carbohydrate metabolism (amylosucrase), and lipid metabolism (NADPH-dependent stearoyl-CoA 9-desaturase). Pup amidohydrolase), carbohydrate metabolism (amylosucrase), and lipid metabolism (NADPH-dependent stearoyl-CoA 9-desaturase), while a small number of KOs are associated with stress. Also, a small number of KOs are associated with stress response or membrane defense (outer membrane immunogenic protein, arginine decarboxylase). In contrast, CC6 and CC6 (RH) soils were enriched in KOs involved in energy metabolism, amino acid degradation, stress response, and membrane-associated defense, with key KOs including H+-transporting ATPase, Pup amidohydrolase, glycine cleavage system P protein, arginine decarboxylase, 3-hydroxy-2-methylpyridine-4,5-dicarboxylate 4-decarboxylase, and outer membrane immunogenic protein. Whereas the heatmap of KOs of fungi (Figure 4d) showed the highest abundance of KOs in CC6 (RH) soil, followed by CC4 (RH). In CC2 soils, KOs abundance fluctuated markedly with high and low levels, but the overall heatmap color was close to neutral (white). The lowest abundance was found in CK soils, while the other two groups, CC6 and CC4, showed moderately low abundance with similar colors. The most abundant fungal KOs included nuclear pore complex protein Nup205, HEAT repeat-containing protein 5, ankyrin repeat domain-containing protein 50, protein-serine/ threonine kinase, DNA-directed RNA polymerase III subunit RPC1, chitin synthase, ferricrocin synthase, vacuolar protein sorting-associated protein 13A/C, and voltage-directed RNA polymerase III subunit RPC1. protein 13A/C, and voltage-dependent calcium channel. These KOs are mainly involved in cell structure and growth, metabolic regulation, and stress/signal sensing functions, reflecting the enhanced fungal activity and adaptive capacity in rhizosphere and long-term soils associated with CCO.

KEGG pathway analysis revealed that continuous stress significantly enriched (CK vs. CC6 or CC6 (RH)) key pathways related to metabolism, signaling, and stress adaptation in bacteria (Figures 4e,f) and fungi (Figures 4g,h). Bacterial enrichment pathways included Nucleocytoplasmic transport, Motor proteins, N-glycan biosynthesis, Phosphatidylinositol signaling, Thyroid function, and Nucleocytoplasmic transport. Phosphatidylinositol signaling, Thyroid hormone signaling, and Regulation of the actin cytoskeleton. Fungal enrichment pathways include Secondary metabolites and Metabolic pathways. Fungal enrichment pathways include Metabolic pathways, Biosynthesis of secondary metabolites, Microbial metabolism in diverse environments, Nucleocytoplasmic transport, the regulation of actin cytoskeleton MAPK signaling pathway—yeast, and Cell cycle and Autophagy—yeast. These pathways jointly regulate intracellular transport, protein modification, microbial growth, and stress response, revealing the coordinated functional adaptations of soil microorganisms to successive stresses.

### 3.5 Intergroup differences and multivariate analysis of soil metabolite composition

**FIGURE 5:**
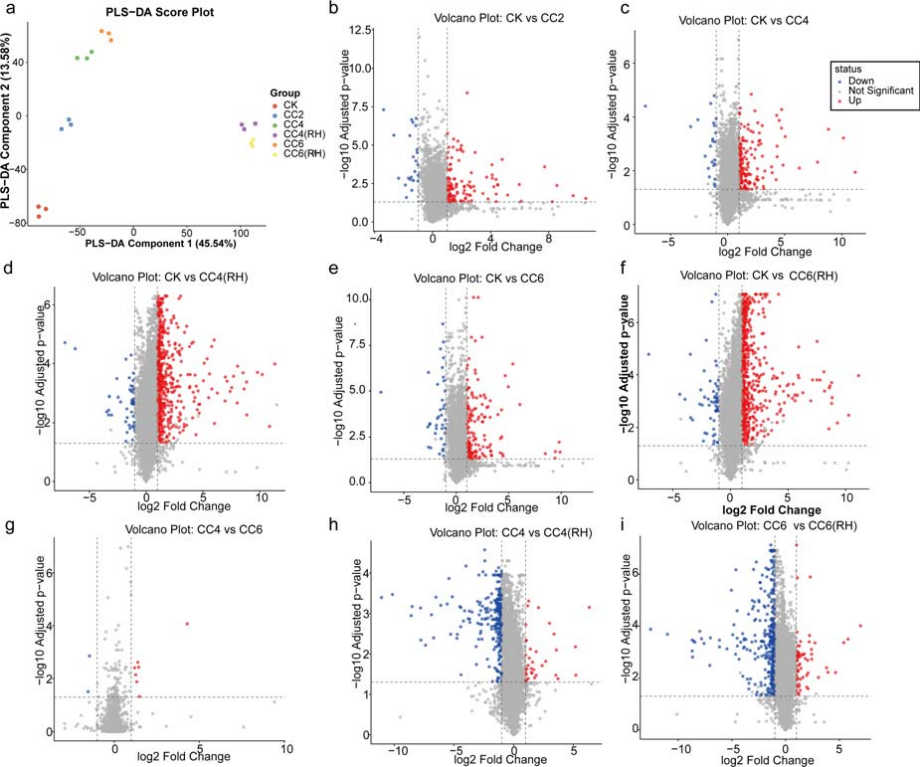
Multivariate and differential analysis of soil metabolomes under continuous cropping. Partial Least Squares Discriminant Analysis (PLS-DA) shows the separation of metabolite profiles among soil samples **(a)**. Volcano plots illustrating differentially abundant metabolites between soil treatments **(b–i)**. CK, control soil without blueberry cropping; CC2, soil under 2-year continuous cropping; CC4, bulk soil under 4-year continuous cropping; CC4 (RH), rhizosphere soil under 4-year continuous cropping; CC6, bulk soil under 6-year continuous cropping; CC6 (RH), rhizosphere soil under 6-year continuous cropping.

Partial Least Squares Discriminant Analysis (PLS-DA) was performed to reveal the relationship between metabolite profiles and soil samples (Figure 5a). The results showed significant separation among soil samples, indicating differences in metabolite abundance among different soils. Through further analysis of variance (Figures 5b–i), we identified 170 metabolites significantly up-regulated, 37 down-regulated, and 14,368 non-significantly different between CK and CC6; 624 metabolites up-regulated, 57 down-regulated, and 13,894 non-significantly different between CK and CC6 (RH); and 397 metabolites significantly up-regulated, 68 down-regulated, and 14,119 non-significantly different between CC6 and CC6 (RH). The comparison between CC6 and CC6 (RH) showed 397 metabolites significantly up-regulated, 68 down-regulated, and 14,119 not significantly different.

### 3.6 Distribution patterns and functional pathways of soil metabolites under different groups

**FIGURE 6:**
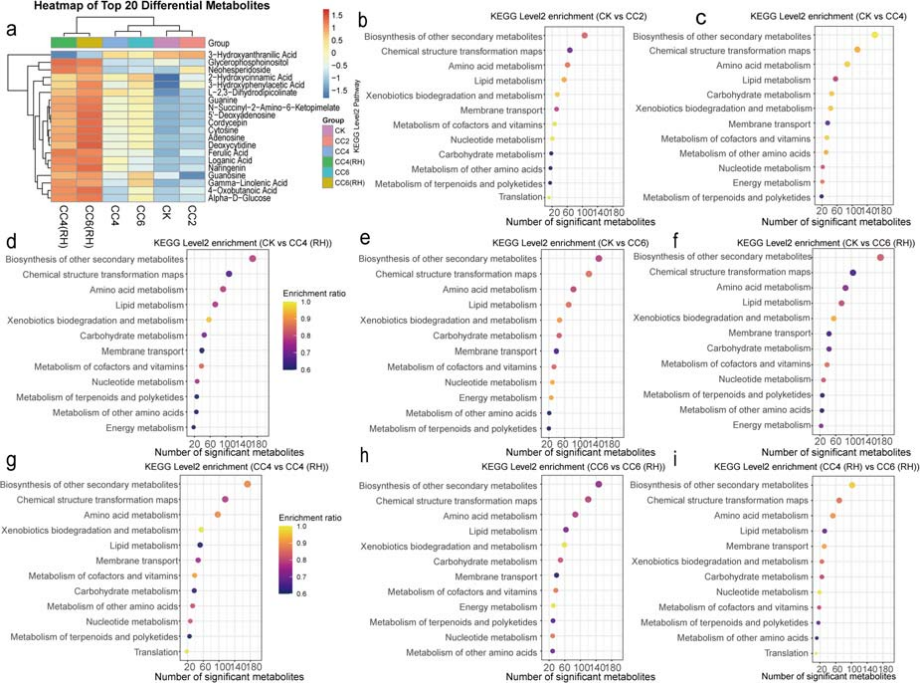
Top differential metabolites and functional pathway enrichment in soil metabolomes. Heatmap depicting the top 20 metabolites exhibiting the most pronounced differences across soil treatments and compartments **(a)**. KEGG Level 2 pathway enrichment analysis illustrating the major metabolic pathways associated with observed variations in soil metabolomes **(b–i)**. CK, control soil without blueberry cropping; CC2, soil under 2-year continuous cropping; CC4, bulk soil under 4-year continuous cropping; CC4 (RH), rhizosphere soil under 4-year continuous cropping; CC6, bulk soil under 6-year continuous cropping; CC6 (RH), rhizosphere soil under 6-year continuous cropping.

The top 20 differential metabolites exhibited distinct distribution patterns across groups and soil compartments (Figure 6a). Most metabolites, including 3-Hydroxyanthranilic acid, Glycerophosphoinositol, Neohesperidoside, 2-Hydroxycinnamic acid, 3-Hydroxyphenylacetic acid, L-2,3-Dihydrodipicolinate, Guanine, N-Succinyl-2-amino-6-ketopimelate, 5′-Deoxyadenosine, Cordycepin, Cytosine, Adenosine, Deoxycytidine, Ferulic acid, Loganic acid, Naringenin, Guanosine, Gamma-linolenic acid, 4-Oxobutanoic acid, and Alpha-D-glucose, were particularly enriched in the rhizosphere of CC6 (RH) soils, suggesting active metabolic accumulation associated with rhizosphere soils processes. In contrast, 3-Hydroxyanthranilic Acid and 4,6-Dinitro-O-Cresol displayed markedly lower abundance in rhizosphere soil groups, with higher levels detected in the CK group, indicating distinct depletion patterns under rhizosphere soils influence.

KEGG Level 2 enrichment analysis (Figures 6b–i) revealed that, except for the comparison between CC4 and CC4 (RH), the top four enriched pathways across all other group comparisons were Biosynthesis of other secondary metabolites, Chemical structure transformation maps, Amino acid metabolism, and Lipid metabolism. According to the volcano plots, the metabolic alterations induced by continuous cropping were primarily concentrated in the rhizosphere soil compartment. In the comparison of CK vs CC4 (RH), besides the four predominant pathways, additional enriched categories—ranked by enrichment degree—were Xenobiotics biodegradation and metabolism, Carbohydrate metabolism, Membrane transport, Metabolism of cofactors and vitamins, Nucleotide metabolism, Metabolism of terpenoids and polyketides, Metabolism of other amino acids, and Energy metabolism. Similarly, for CK vs. CC6 (RH), the enriched pathways in ascending order of enrichment included Xenobiotics biodegradation and metabolism, Membrane transport, Carbohydrate metabolism, Metabolism of cofactors and vitamins, Nucleotide metabolism, Metabolism of terpenoids and polyketides, Metabolism of other amino acids, and Energy metabolism.

## 4 Discussion

### 4.1 Distinct microbial diversity responses between rhizosphere and soil under CCO stress

Continuous cropping obstacles are primarily driven by persistent plant–soil feedback loops mediated by root exudate-induced autotoxic compound accumulation and progressive restructuring of rhizosphere microbial communities. Although agronomic interventions may alleviate symptom severity, they are generally insufficient to disrupt this feedback system due to its intrinsic nature within plant–soil–microbe interactions (Haq et al., 2025).

Our study showed an overall decline in bacterial and fungal diversity in soils after continuous blueberry planting, suggesting that continuous monocropping has a broadly inhibitory effect on soil microbial communities. At the spatial scale, there were significant differences in the response of different soil microbial taxa to continuous blueberry cropping: bacterial diversity declined mainly in non-rhizosphere soils, whereas fungal diversity was more sensitive to the rhizosphere environment, and its decline was mainly concentrated in rhizosphere soils. This suggests an often-overlooked issue: soil improvement in continuous cropping should be stratified, with bacterial problems focusing on overall soil improvement and fungal problems focusing on the rhizosphere environment, and the choice of improvement methods should be more strategic rather than blindly trying out a variety of tools (Li et al., 2024). We also observed that the rhizosphere fungal community was significantly affected even in early CCO soils, and it can be inferred that the blueberry root system and its microenvironment may be the main driver of this change (Liu C. et al., 2024). Interestingly, the difference in microbial diversity between rhizosphere and non-rhizosphere soils remained significant at 4 years of continuous cropping, but disappeared after 6 years of continuous cropping. The study suggests that this “convergence” is not due to an increase in diversity caused by a weakening of the rhizosphere screening effect, but instead is associated with a decrease in the diversity of non-rhizosphere soil microorganisms. This pattern may indicate that long-term continuous cropping has weakened the non-rhizosphere microbial community, thereby reducing the difference between rhizosphere and non-rhizosphere soils. Consequently, this narrowing may reduce the functional redundancy that non-rhizosphere soils provide as background soils (Li et al., 2021), and the variety of soil microorganisms that can be recruited and enriched by rhizosphere soils (Chen et al., 2023), which greatly reduces crop resistance to mitigating shocks, and potentially makes crops more susceptible to the effects of CCO.

Consistent with the results of α-diversity analysis, PCoA analysis further validated the differential response of bacteria and fungi in rhizosphere and non-rhizosphere soils at the β-diversity level. The changes in bacterial community structure were mainly reflected in the non-rhizosphere soil, while the fungal community structure was mainly driven by the rhizosphere environment, which further indicated that there were significant differences in the spatial scales of the response mechanisms of different microbial groups in the blueberry continuous cropping process.

### 4.2 Effects of continuous blueberry cropping on soil bacterial and fungal community composition

Microbial community analyses revealed that long-term blueberry continuous cropping significantly stronger effects on fungal communities than on bacterial communities at the phylum and genus levels, suggesting that fungi may play a more critical role in the rhizosphere ecological changes associated with CCO. Notably, these microbial changes were mainly concentrated in rhizosphere soils, highlighting the role of the rhizosphere as a central ecological interface where long-term continuous cropping affects soil function and plant growth performance. This is consistent with findings from previous studies on other crops, which suggest that continuous cropping may lead to negative effects in the rhizosphere (Wu et al., 2024).

Among the bacterial communities, healthy CK soils were mainly enriched with taxa closely related to nutrient cycling and soil functional stability, such as Actinomycetota and Nitrososphaerota, many members of which are known to promote plant growth and suppress soil-borne pathogens (Bhatti et al., 2017; Zhao et al., 2023). In contrast, six consecutive years of continuous blueberry cropping significantly enriched acidophilic bacterial taxa such as Verrucomicrobiota and *Candidatus Acidoferrales*. The increase in these taxa, which are often considered indicators of acidic and nutrient-poor environments, suggests that long-term blueberry cultivation may exacerbate rhizosphere soil acidification and reduce nutrient availability (Epihov et al., 2021; Chen et al., 2025). This judgment is also supported by changes in fungal communities, such as the significant reduction or even absence of Sordariomycetes (Xing et al., 2022), which is negatively correlated with soil acidity, in the rhizosphere soils of CC6 (RH), further suggesting that long-term continuous cropping has caused the rhizosphere environments to progressively deviate from the range of suitability for blueberry growth. Meanwhile, Lecanoromycetes are commonly associated with drought and nutrient-poor habitats (Hustad et al., 2013; Eldridge et al., 2015; Choe et al., 2021), and their occurrence in CC6 (RH) implies that long-term continuous cropping may cause both nutrient limitation and water stress at the rhizosphere scale.

At the level of fungal communities, CC6 (RH) soils showed significant functional structural remodeling. Mutualistic symbiosis and nutrient acquisition-related fungal taxa, especially Mucoromycota, were significantly reduced compared to CK soils. This group usually exists as fine-rooted endophytic fungi with ecological functions similar to those of tufted mycorrhizal fungi, which contribute to the uptake of inorganic and organic nutrients by plant roots (Prout et al., 2024). Thus, its decrease suggests that plant–fungal mutualisms may be weakened under prolonged continuous cropping stress, with a reduced capacity for rhizosphere nutrient acquisition. Instead, saprophytic and potentially pathogenic fungi gradually dominated. The enrichment of Ascomycota and Basidiomycota reflected the enhanced capacity of rhizosphere organic matter decomposition and root secretion utilization, but these taxa also contained a large number of plant pathogenic fungi, such as the genus *Fusarium*, as well as specialized parasitic fungi, such as rusts and black powder fungi (Kjøller and Rosendahl, 2014; Challacombe et al., 2019). In addition, the enrichment of saprophytic-dominant Geoglossomycetes indicates the accumulation of senescent or decaying root residues in the rhizosphere zone, which provides a favorable ecological niche for saprophytic fungi.

This functional shift was further corroborated at the genus level, where the enrichment of *Serendipita* in rhizosphere soils of successive crops may reflect the increased dependence of plants on symbiotic fungi with pro- and antiseptic functions under long-term stress conditions (Cheng et al., 2020; González Ortega-Villaizán et al., 2024). In contrast, increases in the saprophytic fungi *Geoglossum* and *Trichoglossum* indicate an increase in the proportion of decaying root residues in the rhizosphere (Hustad et al., 2013). At the same time, the decreases of *Aspergillus* and *Rhizopus* may have weakened the ability of the soil to buffer the stress of continuous cropping, and the decreases of *Aspergillus*, which plays an important role in promoting plant growth and suppressing pathogens, and *Rhizopus*, which is mainly involved in the decomposition of organic residues, may be related to the gradual lignification of the rhizosphere residues or the increase of the competition for ecological niches in the long term continuous cropping conditions (Hung and Lee Rutgers, 2016; Daigham et al., 2024). It is worth noting that the microbial community structure of CC4 soils is more likely to represent a “transition state” rather than a fully imbalanced rhizosphere system, and that the enrichment of CC4 soils with Rhodospirillales and Sphingomonadales may help to maintain the stability of the microbial network in acidic environments, thus providing some protection to soil health (Chen et al., 2014; Huang et al., 2018; Zhou et al., 2024). Among fungal communities, enrichment of CC4 (RH) soils with Sebacinales and Leotiomycetes may enhance plant adaptation to pathogenic stress and adverse environmental conditions (Nguyen and Seifert, 2008; Martino et al., 2018; Tian et al., 2024). This microbial structure may have buffered the negative effects of continuous cropping to some extent, which could explain why no obvious differences in plant growth and health were observed in CC4 soils, despite clear signals of microbial community shifts. However, this inference requires further validation with direct plant performance data.

In contrast, the absence of these potentially “protective” microbial groups and the dominance of saprophytic and potentially pathogenic fungi in CC6 (RH) soils suggests that the rhizosphere microbial system has shifted from a buffered transition state to an imbalanced state. Overall, the long-term continuous blueberry cropping contributed to the transformation of the rhizosphere microbial community from a relatively functionally balanced state dominated by mutualistic symbioses to an acidified, nutrient-limited structure dominated by saprophytic and potentially pathogenic microbes, and this functional remodeling is likely to be an important microbiological mechanism for the development of the CCO.

### 4.3 Functional implications of microbial community changes under continuous blueberry cropping

Microbial functional analyses further confirmed the trend of systematic changes in soil microbial community structure under long-term blueberry continuous cropping conditions. Although the bacterial functional genes that differed significantly between CK and CC6 were mainly focused on metabolism-related processes and showed strong functional redundancy overall, the significant changes in their abundance patterns suggest that the main performers of key metabolic functions were replaced during successive cropping, rather than a simple loss of overall metabolic potential. This functional-level turnover implies that similar biochemical processes can still be maintained during different stages of continuous cropping, but their operational efficiency may be altered by changes in the dominant microbial taxa.

It is noteworthy that bacterial functional profiles in CC6 soils were significantly skewed toward environmental stress-related processes, especially those related to acid stress response and ion homeostasis regulation; in contrast, CK soils were enriched with more core metabolic functions involved in amino acid metabolism and nitrogen cycling. This trend of functional differentiation is highly consistent with the inference that soil acidification is caused by long-term continuous cropping, indicating that the functional focus of the soil microbial system has gradually shifted from highly efficient nutrient metabolism to a state dominated by environmental stress adaptation. In contrast, the functional characteristics of CC4 were intermediate between those of CK and CC6, suggesting that it was still in the transition stage from stabilization to imbalance, which might be an important reason why it had not yet shown obvious CCO at this stage.

In fungal communities, the functional differences between CK and CC6 (RH) are mainly reflected in cell wall synthesis, transcription and signaling regulation, and transmembrane transport. From an ecological point of view, these functional changes as a whole reflect the enhanced metabolic activities of eukaryotic microorganisms in the context of continuous cropping stress. However, in conjunction with the changes in fungal community composition, it can be further inferred that this apparent functional enhancement is more likely to stem from the expansion of saprophytic and potentially pathogenic fungi than from the improved functioning of symbiotic fungi. Meanwhile, the activation of transcription and signal regulation-related functions may reflect the stress response and defense mechanisms initiated by some fungal taxa under unfavorable environmental conditions.

Consistent with the above results, KEGG tertiary pathway analysis showed that the bacterial “Biosynthesis of ansamycins” pathway was significantly enriched in CC6. As a functional pathway related to antibiotic synthesis, its enhancement may indicate that microorganisms synthesize antibiotic-like secondary metabolites to cope with the increasing ecological pressures under long-term successive cropping stresses and increased competition and antagonism within the soil microbial community.

Taken together, the synergistic changes in bacterial and fungal communities at the functional level suggest that long-term continuous blueberry cropping drives the soil microbial system toward a state characterized by stress adaptation but with overall reduced metabolic efficiency, thus laying an important microbiological foundation for the formation of CCO.

### 4.4 Metabolite profile shifts in rhizosphere soils during continuous cropping

Among the significantly enriched metabolic pathways, “biosynthesis of other secondary metabolites” consistently showed the strongest response under long-term continuous cropping conditions in blueberry, suggesting that secondary metabolism is an important biochemical interface connecting the remodeling of rhizosphere microbial structure and plant stress response. Secondary metabolites are widely recognized as key mediators of plant-microbe interactions, and their accumulation is highly sensitive to changes in root secretion patterns and microbial community composition during long-term continuous cropping (Gross, 2009; Hickman et al., 2021; Yuan et al., 2022; Haq et al., 2025). Although phenolic acids are often recognized as important chemical drivers of CCO (Ma H. Y. et al., 2023; Zhou et al., 2025), of the first 20 differential metabolites identified in this study, only ferulic acid, 3-hydroxyphenylacetic acid, and 2-hydroxycinnamic acid were strictly classified as phenolic acids. This result suggests that long-term continuous cropping of blueberries is more likely to induce the selective accumulation of specific phenolic acid derivatives rather than extensive enrichment of traditional phenolic acids. These findings indicate that selective accumulation of specific phenolic acid derivatives rather than extensive enrichment of traditional phenolic acids is more likely to occur during continuous blueberry cropping.

Ferulic acid is a phenolic acid typically closely associated with CCO and has been widely reported in a variety of crop continuous cropping systems. Numerous studies have shown that high concentrations of ferulic acid in the rhizosphere can inhibit seed germination and root elongation (Devi and Prasad, 1992), induce oxidative stress (Wu et al., 2018), and reshape the structure of the rhizosphere microbial community (Zhao et al., 2015; Li et al., 2016), thereby triggering a CCO. In this study, ferulic acid is the most well-studied representative compound of the three phenolic acid differential metabolites, and thus has a clear theoretical potential to trigger blueberry CCO. However, although ferulic acid was detected as one of the differential metabolites, its abundance was not the most significant, and it accumulated to a lower extent compared to 3-hydroxyphenylacetic acid and 2-hydroxycinnamic acid. The autotoxic effect of phenolic acids is usually concentration-dependent (Li et al., 1993), and metabolomics data reflect relative abundance rather than absolute concentration. Therefore, based on the available data, it is not yet possible to directly determine whether they play a dominant role in the formation of blueberry succession disorder, which still needs to be further clarified in combination with absolute quantitative analysis and biological validation experiments.

To date, 3-hydroxyphenylacetic acid has rarely been reported in studies related to CCO or chemosensory autotoxicity. The current evidence focuses on the positional isomer p-hydroxyphenylacetic acid, which has been shown to inhibit the growth of legumes, but its inhibitory effect is weaker than that of the common hydroxybenzoic acids (Asaduzzaman and Asao, 2012). Despite their high structural similarity, different substitution positions may lead to significantly different biological activities. p-Hydroxyphenylacetic acid is usually derived from the downstream metabolism of L-tyrosine (Sparnins and Chapman, 1976), whereas the high accumulation of 3-hydroxyphenylacetic acid at the meta position in this study suggests that it may be derived from meta-tyrosine. Given that the formation of meta-tyrosine is usually closely related to oxidative stress (Ipson and Fisher, 2016), the significant accumulation of 3-hydroxyphenylacetic acid in this study may indicates, to some extent, that strong oxidative stress may exist in the rhizosphere environment of blueberry under long-term continuous cropping conditions. However, the specific biosynthesis sources of this compound and its ecological functions in the continuous cropping system still need to be further elucidated. Regardless of the specific biosynthetic pathway, the high abundance of 3-hydroxyphenylacetic acid suggests that it may be a previously overlooked potential chemosensitizer in blueberry continuous cropping systems.

Similarly, 2-hydroxycinnamic acid, a derivative of cinnamic acid, has received less attention in research on CCO, but cinnamic acid itself has been widely demonstrated to have significant chemosensory autotoxic effects (Liu et al., 2010; Guo et al., 2020). The significant enrichment of 2-hydroxycinnamic acid in blueberry rhizosphere soils in this study is consistent with the established knowledge of the presence of oxidative stress under long-term continuous cropping conditions. It has been shown that 2-hydroxycinnamic acid can be involved in reactive oxygen species scavenging processes (Jia et al., 2024), and its accumulation may represent part of a rhizosphere oxidative stress response process co-mediated by plants or microorganisms under long-term continuous cropping conditions. Meanwhile, given its structural relevance to cinnamic acid, 2-hydroxycinnamic acid may also have potential chemosensory or growth inhibitory effects, but further experimental verification is needed (Garcia-Jimenez et al., 2018).

Overall, the metabolomics results suggest that long-term continuous blueberry cropping induced a synergistic shift in the rhizosphere metabolic profile characterized by selective accumulation of stress-related secondary metabolites. Combined with the results of microbial community structural remodeling and functional shifts toward stress adaptation, these metabolic changes likely represent a chemical characterization of a stress-adapted but functionally limited rhizosphere system and play an important role in the development of the CCO. Enrichment of ferulic acid, 3-hydroxyphenylacetic acid, and 2-hydroxycinnamic acid, combined with evidence of oxidative stress in the rhizosphere, suggests that metabolic stress signaling and chemosensory effects may play an important role in the development of blueberry CCO.

### 4.5 Limitations and perspectives

Although this study provided a comprehensive analysis of the potential microbial and metabolic mechanisms underlying blueberry CCO using metagenomic and metabolomic data, some limitations remain. First, our analysis was primarily based on correlations and lacked controlled experiments or functional validation, such as pot or field trials, to confirm the causal roles of key microbial groups or metabolic pathways in continuous cropping stress. This limits the depth of mechanistic interpretation. In addition, all samples were collected at a single time point, which may be affected by seasonal or temporal variations. Another limitation of this study is that all samples were collected from a single site. However, previous studies have shown that even within the same crop species, continuous cropping effects on soil physicochemical properties and microbial communities can vary across different regions. Therefore, site-specific effects cannot be excluded in this study (Han et al., 2025). Multi-time-point or long-term sampling would better capture the temporal dynamics of microbial communities and metabolites, offering a more reliable assessment of the long-term effects of continuous cultivation.

Moreover, this study did not systematically evaluate the influence of agricultural practices, such as fertilization regimes, on the development of CCO. Previous work has shown that long-term improper fertilization—particularly excessive nitrogen application—can exacerbate continuous cropping stress by lowering soil pH (Song et al., 2022), altering microbial community structure, and suppressing beneficial microbes (Zhang et al., 2022). Given that we also observed soil acidification and microbial imbalance, it is possible that fertilization practices contributed, at least in part, to the patterns seen in microbial and metabolite profiles.

From a practical perspective, this study offers valuable insights for managing blueberry CCO systems. The changes in microbial communities and fluctuations in metabolites associated with CCO that we identified could potentially serve as indicators for monitoring soil health. For example, certain microbial groups are significantly enriched in CCO soils, while key metabolic pathways are disrupted—together, these two characteristics could potentially be used to establish early warning signals for CCO stress. Furthermore, these findings provide a basis for designing targeted management strategies, such as the application of microbial inoculants, bio-organic fertilizers, or approaches aimed at improving soil function by modulating the rhizosphere microenvironment, ultimately helping to maintain blueberry yield stability.

Despite these limitations, our study systematically examined how different durations of continuous cropping affect blueberry soil and rhizosphere microbial communities and metabolic profiles, providing a basis for understanding CCO-related stress. Future research could further quantify the effects of continuous cropping on blueberry yield, assess the development of plant diseases, and explore how fertilization and other soil management strategies influence soil health. In addition, the microbial and metabolic changes revealed here could help guide targeted management strategies, such as the use of biofertilizers (Xu et al., 2024), optimized nutrient management, or improvements to the rhizosphere environment, to mitigate CCO and enhance both plant growth and soil sustainability.

## 5 Conclusion

Our results suggest that continuous cropping may drive a synergistic remodeling of the structure, function, and metabolic networks of the rhizosphere microbial community. The resulting shift from a “biotrophic” to a “stress-tolerant” functional state may weaken the functional stability and resilience of the soil system. Such microbial changes could be linked to the risk of blueberry yield decline under continuous cropping, although direct evidence from plant growth and yield measurements is still needed. Furthermore, our findings highlight the potential importance of the rhizosphere microenvironment in crop performance, providing a scientific basis for future studies on soil health regulation and strategies to mitigate cropping obstacles in blueberry orchards.

## Data Availability

The datasets presented in this study can be found in online repositories. The names of the repository/repositories and accession number(s) can be found in the article/Supplementary material.

## Footnotes

1
https://github.com/OpenGene/fastp

2
https://github.com/voutcn/megahit

3
https://github.com/hyattpd/Prodiga

4
http://weizhongli-lab.org/cd-hit

5
https://github.com/ShujiaHuang/SOAPaligner

6
http://ab.inf.uni-tuebingen.de/software/diamond/

7
http://www.hmdb.ca

8
https://metlin.scripps.edu/

9
https://cloud.majorbio.com
