# Supplementary material for: Multi-omics reveal soil microbial dysbiosis and metabolite toxicity as drivers of blueberry continuous cropping obstacles
Source: Front Microbiol. 2026 Jun 26;17:1880203. doi: 10.3389/fmicb.2026.1880203 (PMC13350179; doi:10.3389/fmicb.2026.1880203)
Supplement: Supplementary file 1 [file Table_1.docx]

**Supplementary Material**

**Table S1**

| Group | AN (mg·kg⁻¹) | AP (mg·kg⁻¹) | SOC (%) | EC (μS/cm) | pH |
| --- | --- | --- | --- | --- | --- |
| CK | 58.91 | 25.8876 | 20.48 | 30.9 | 6.62 |
| CC2 | 79.7 | 7.4052 | 27.84 | 52.53 | 5.41 |
| CC4 | 100.49 | 14.8728 | 46.96 | 63.27 | 4.88 |
| CC4(RH) | 121.28 | 17.7715 | 54.86 | 79.57 | 4.73 |
| CC6 | 131.67 | 16.764 | 48.6 | 52.17 | 4.69 |
| CC6(RH) | 145.53 | 13.346 | 55.1 | 65.13 | 4.38 |

Basic physical and chemical properties of soil

Note: CK, control soil without blueberry cropping; CC2, soil under 2-year continuous cropping; CC4, bulk soil under 4-year continuous cropping; CC4 (RH), rhizosphere soil under 4-year continuous cropping; CC6, bulk soil under 6-year continuous cropping; CC6 (RH), rhizosphere soil under 6-year continuous cropping.

AN = available nitrogen; AP = available phosphorus; SOC = soil organic carbon; EC = electrical conductivity; pH = soil pH
